# Coordination Between the Sexes Constrains the Optimization of Reproductive Timing in Honey Bee Colonies

**DOI:** 10.1038/s41598-017-02878-8

**Published:** 2017-06-01

**Authors:** Natalie J. Lemanski, Nina H. Fefferman

**Affiliations:** 1 Department of Ecology, Evolution, and Natural Resources, Rutgers University, New Brunswick, New Jersey, United States of America; 2 Department of Ecology and Evolutionary Biology, University of Tennessee, Knoxville, Tennessee, United States of America

## Abstract

Honeybees are an excellent model system for examining how trade-offs shape reproductive timing in organisms with seasonal environments. Honeybee colonies reproduce two ways: producing swarms comprising a queen and thousands of workers or producing males (drones). There is an energetic trade-off between producing workers, which contribute to colony growth, and drones, which contribute only to reproduction. The timing of drone production therefore determines both the drones’ likelihood of mating and when colonies reach sufficient size to swarm. Using a linear programming model, we ask when a colony should produce drones and swarms to maximize reproductive success. We find the optimal behavior for each colony is to produce all drones prior to swarming, an impossible solution on a population scale because queens and drones would never co-occur. Reproductive timing is therefore not solely determined by energetic trade-offs but by the game theoretic problem of coordinating the production of reproductives among colonies.

## Introduction

All organisms must make trade-offs in how they allocate limited resources among growth, maintenance, and reproduction. The seasonal availability of many critical resources, such as food or habitat, further complicate these trade-offs. In addition, the total energy budget is not static through time; allocating more energy to growth at one point in time means more total energy available in the future, but at the expense of immediate investment in reproduction.

Eusocial insects, such as the European honey bee (*Apis mellifera*) are a good model system for studying these trade-offs. Honey bees live in colonies composed of a single reproductive female and thousands of functionally sterile workers^1^. The colony has a shared energy budget and mostly shared reproductive interests^2, 3^. Honey bee societies comprise three morphological castes: workers, queens, and drones. Investment in each of these castes can conveniently represent the different methods of allocating limited resources toward different life history requirements. Workers contribute to colony growth and survival by performing all foraging, brood care, and defense^1^. The queen’s sole job is to lay eggs; the colony rears new queens only when the colony is ready to reproduce or when the original queen requires replacement^1, 3^. Drones are the male reproductives of a honey bee colony. The drones consume colony resources and perform no work; their sole purpose is to pass on the colony’s genes by mating with queens from other colonies^1^. Thus, workers typically eject drones from the colony in early fall once they have little chance of mating^1^.

Honey bees, along with army ants^4, 5^, stingless bees^6^, and some social wasps^7^, form new colonies by swarming, a process in which a new queen is reared and the entire colony fissions, with a fraction of the worker population going with the old queen (called the prime swarm) and the rest remaining in the original colony with the new queen. While drones can be considered an investment in reproduction, workers are therefore an investment in both growth and reproduction. Previous theoretical work on the timing of reproductive investment in annual social insect colonies has found there is an optimal time to switch from pure growth to pure reproduction to maximize the number of reproductives produced, otherwise called a “bang-bang strategy”^8–10^. However, because the swarm is a large part of the colony’s investment in queens, the annual reproductive success of a honeybee colony can more appropriately be considered as the total of all surviving colonies related to the parent colony: the original colony headed by a daughter queen, the prime swarm headed by the original queen, afterswarms headed by daughter queens, and any colonies fathered by the parent colony’s drones.

In the swarm-founding social insects, there has been much work on optimal sex ratio^11, 12^, swarm fraction^13, 14^, and queen choice^15^, but very little on reproductive timing. The timing of investment in reproduction is extremely important to reproductive success. Temperate honey bee colonies persist year-round, but their food supply is only present in spring and summer when flowers are in bloom^16^. Thus, colonies have a limited period each year in which to grow, reproduce, and store enough food to survive winter.

Because of the dynamics of exponential growth, investing directly in reproduction (via production of drones) rather than growth (via production of workers) early in the year, leads to a smaller final colony size than could be achieved by investment in growth first and reproduction later in the year. Because each worker produced contributes to the production of future workers (i.e. an investment with compounding interest), investing in drones early siphons resources away from that capital and thereby reduces the energy available to produce either drones or workers later. Since swarming is regulated at a whole-colony level, and colonies must attain sufficient size to swarm, the level of investment in drones versus workers determines the amount of energy the colony can later invest in both swarms and drones, as well as when swarming will occur. The timing of drone production is therefore important in driving both male and female components of reproductive success.

In addition to affecting the total energy budget, the timing of reproductive investment also affects drones’ chances of mating. If mated successfully, a drone can be expected to be the father of approximately 1/12th of the workers and daughter queens of a new colony^17^. However, because there are many times more drones than queens in the population, each individual drone is very unlikely to mate successfully^17^. A drone’s reproductive success depends on the supply of queens with which to mate, so drones should be supported only when other colonies are making swarms, regardless of what would be the best time to produce them based on a colony’s energetic trade-offs between reproduction and somatic growth. Swarms are only successful when there are sufficient drones available to mate with the virgin queen; however, because there are many more drones than queens, almost all virgin queens in a population mate successfully. A new queen’s reproductive success is mostly driven by how large her worker population is and how long the colony has to gather enough resources to survive winter.

We expect all colonies in a population to evolve to produce drones and swarms at the time that maximizes their own reproductive success. However, the best time for each colony to produce male and female reproductives will depend on when other colonies reproduce, as well as the energetic trade-off between growth and reproduction. If the optimal timing of drone production based on colony growth dynamics results in each colony producing drones and queens at the same time, we should expect all colonies to produce drones at the time that both maximizes the drones’ mating success and maximizes colony growth. If the optimal time for a colony to produce drones differs from the optimal time for a colony to produce queens, there is a potential game theoretic conflict among colonies. Each colony would maximize its fitness by producing drones at the energetically optimal time if other colonies produce queens at that time, but all other colonies would also prefer to produce drones at that time as well. Since colonies need to produce drones when other colonies produce queens and vice versa, all colonies cannot produce drones at the energetically optimal time because that would lead to the worst fitness option, failure to coordinate among colonies.

To fully understand the dynamics of this system, we present a mathematical model of honey bee swarm and drone production addressing two questions: a) when should a honey bee colony produce and eject drones to optimize its yearly reproductive success? and b) how does the energetic trade-off between growth and drone production interact with the drone mating success in shaping the timing of colony reproduction?

To answer our questions, we have constructed a linear programming model^18^ of the timing of swarming and drone production in a temperate honey bee colony. Linear programming is a mathematical tool for finding optimal solutions in a system with many interacting variables, which, to the best of our knowledge, has not been previously applied to the question of reproductive timing in insect societies. An advantage of this approach to the question of reproductive timing is that, unlike most previous work^12^, it does not require us to determine what fraction of investment in workers should be counted as an investment in the swarm and what should be counted as an investment in drones. In our model, the objective function to be maximized is annual colony fitness and the constraints involve a limited energy budget allocated among workers, drones, and food stores. The outputs of the model we are most interested in are the optimal time for the colony to invest in daughter queens, via swarming, and the optimal times for the colony to invest in drones.

We also compared two alternative scenarios in the model. In Scenario 1, we calculate the optimal pattern of drone and worker production for a focal colony, assuming other colonies in the population do not change their reproductive behavior in response to the selection pressure we calculate. Instead we assume that queens are available for the focal colony to mate with on a fixed day, regardless of whether it is the optimal time to produce queens.

In Scenario 2, we calculate the optimal pattern of drone and worker production for a focal colony, assuming all colonies in the population produce queens on the optimal day. The purpose of this scenario is to examine how the need to coordinate reproductive timing with other colonies changes the optimal behavior. Realistically, we would not really expect all colonies to produce swarms and drones on the same day because of natural variation in the strength and growth rate of colonies; this variation may partly alleviate a potential conflict between colonies. However, our model ignores inter-colony variability to examine the strongest version of the potential conflict to better understand its effects on reproductive timing.

## Results

### Scenario 1

**Figure 1:**
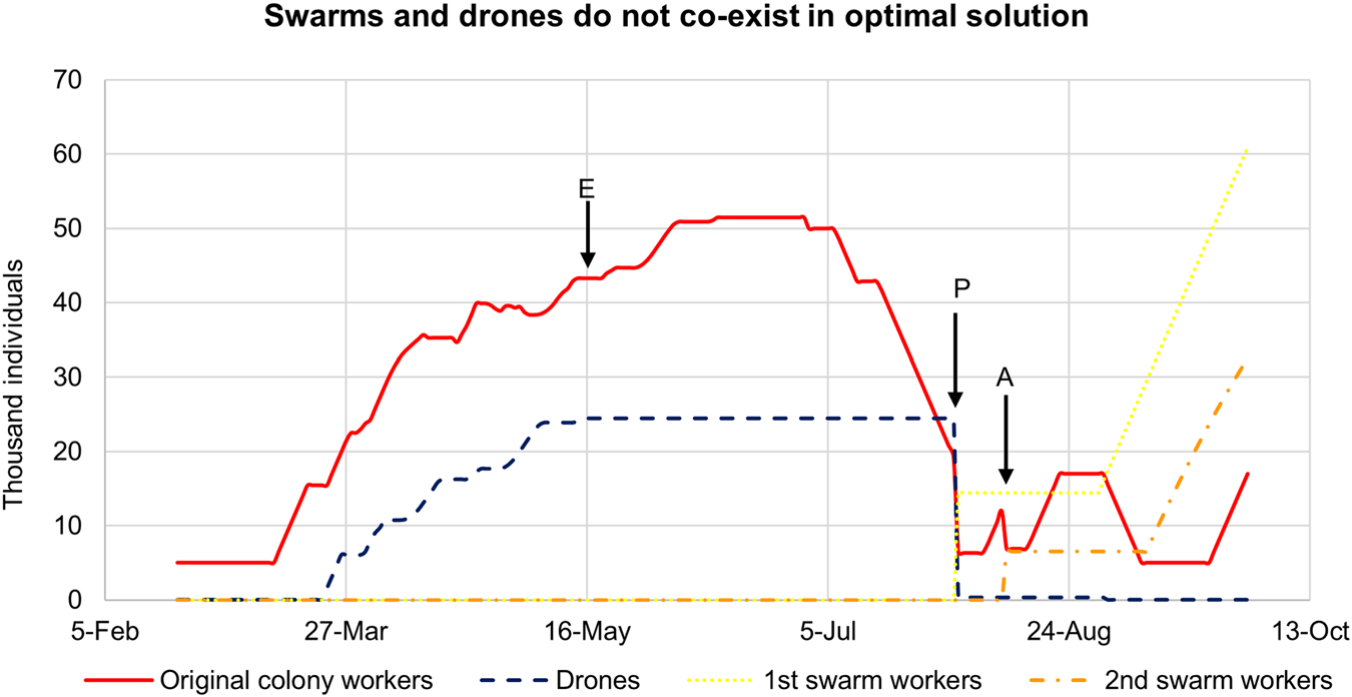
Queen and drone production do not co-occur in optimal solution. The dashed line shows the optimal number of drones and the solid line shows the optimal number of workers present in the colony on each day under model scenario 1. Under the assumption that other colonies in the population produce queens at an empirically estimated time^19^ regardless of whether it is optimal, we find the optimal swarm date for the focal colony to be the end of July. In this model scenario, the optimal behavior is for the colony to produce all drones between April and July and evict all drones before the colony swarms at the end of July. The dotted line shows the number of workers in the prime swarm on each day; the dash-dot line shows the number of workers in the afterswarm on each day in the optimal solution. The letters P and A indicate when the prime swarm and afterswarm, respectively, are produced in the model. The letter E indicates the empirically measured swarm date in mid-May^19^, much earlier than predicted by the model.

**Figure 2:**
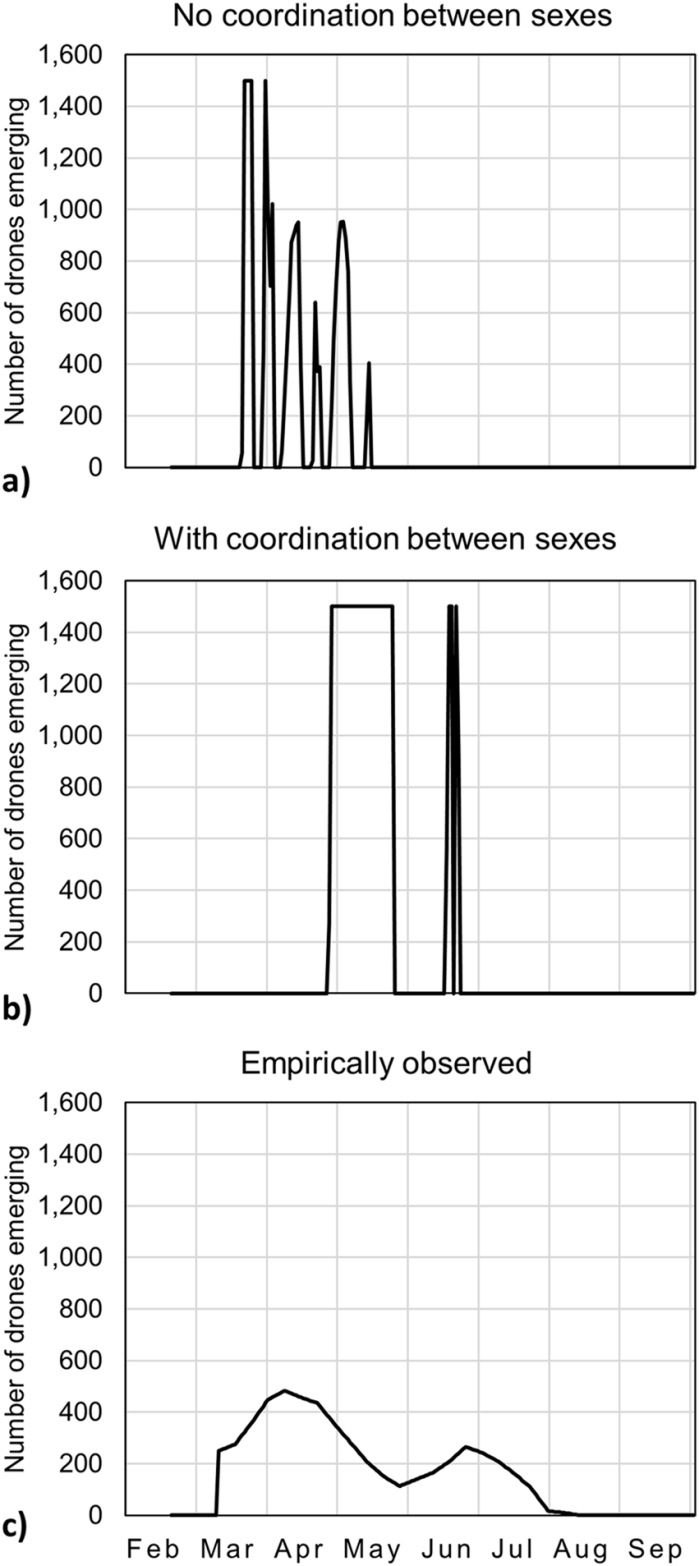
The timing of drone emergence as predicted by the model vs. empirically observed. (**a**) Under model scenario 1, we assume that all other colonies in the population produce queens at an empirically estimated time^19^. We find the optimal solution is for the parent colony to produce all drones early in the spring between March and May, earlier than empirically observed. (**b**) Under model scenario 2, we assume that all colonies in the population produce queens and swarm on the same days as the focal colony; in other words, all colonies follow the same optimal timing. Under this assumption, the optimal solution is to produce a first pulse of drones from late April to late May and produce a second pulse of drones in June. (**c**) The bottom panel shows the empirically observed number of drones emerging each day^19^. Model scenario 2 predicts that colonies should start producing drones later and stop producing drones earlier than empirically observed but captures the empirically observed pattern of two distinct peaks in drone emergence.

Under the assumption that drone mating success is independent of when swarms are produced by the focal colony, we find that, over a broad range of parameters (Supplementary Figs S2–S8), swarms and drones do not co-occur in the optimal solution (Fig. 1). The globally optimal solution is for colonies to produce all drones early in summer and eject all drones prior to swarming (Fig. 1). In addition, the optimal swarm date is late July/early August, much later than the empirically observed average swarm date (May) in the dataset the model parameters were based on (Fig. 1). The model also predicts that all drones should emerge in May and June, rather than continue to emerge into August as they do in empirically observed data (Fig. 2).

### Scenario 2

**Figure 3:**
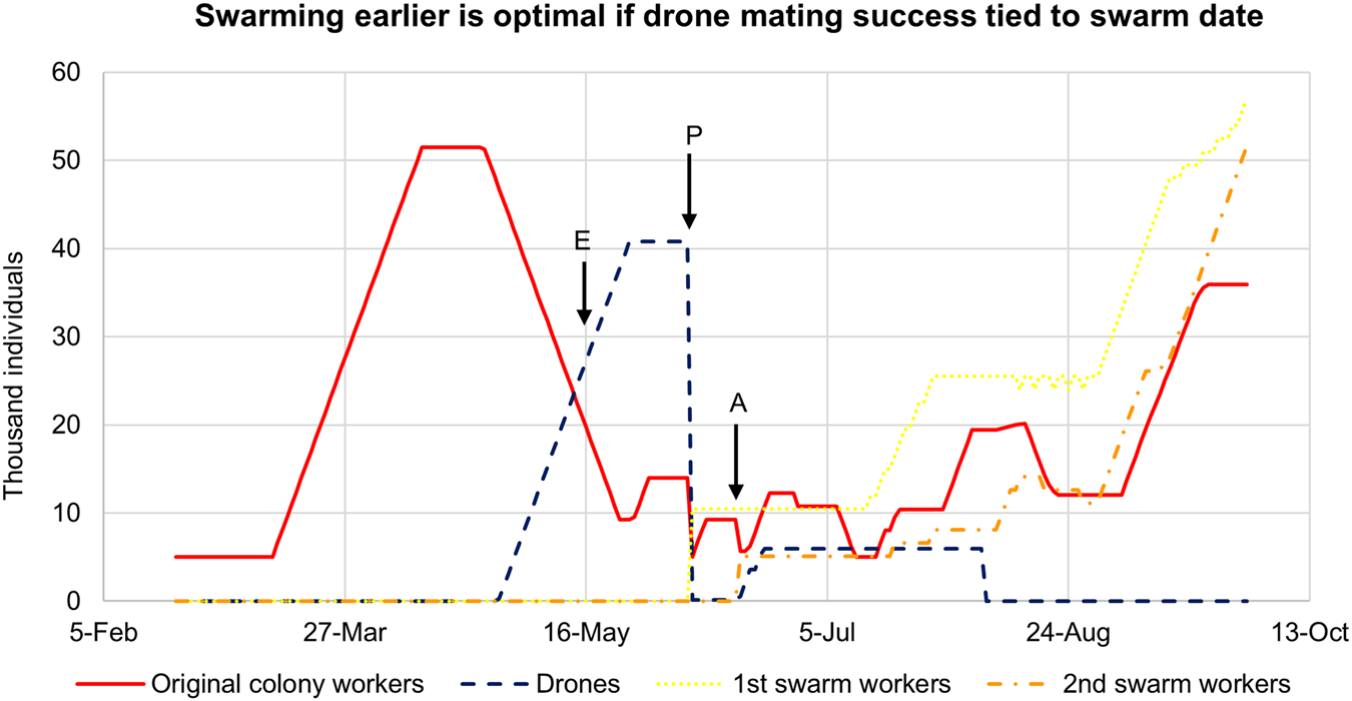
Swarming earlier is optimal if drone mating success is tied to swarm date. In model scenario 2, we assume that all colonies in the population adopt the same optimal timing of drone and swarm production, i.e. the focal colony optimizes the timing of drone and swarm production given that all colonies in the population produce queens and swarm at the same time as the focal colony. Under this set of assumptions, we find the optimal swarm date is in late May/early June and the optimal behavior is to maintain drones from April to late May, evict all drones just before swarming, and maintain a second wave of drones from June to August. The solid line shows the optimal number of workers in the parent colony on each day, the dashed line shows the optimal number of drones on each day, the dotted line shows the number of workers in the prime swarm and the dash-dot line shows the number of workers in the afterswarm. The letters P and A indicate when the prime swarm and afterswarm, respectively, are produced in the model. The letter E indicates the empirically measured swarm date^19^. The optimal swarm date predicted by model scenario 2 is much closer to the empirically observed date than that predicted by model scenario 1.

When we allow drone mating success to depend on the swarm date of the focal colony, we find that drone production and swarm timing in the focal colony overlap (Fig. 3). Colonies are also predicted to swarm earlier, in May, closer to the time of swarming observed in empirical data (Fig. 3). The model predicts two peaks in drone emergence, one before and one after swarming (Fig. 2). Thus, we find that the constraint of requiring coordination between sexes changes the optimal timing of reproductive investment in temperate honey bees.

## Discussion

Our results demonstrate that when the behavior of other colonies is assumed to follow current observed patterns for the timing of swarm and drone production, the optimal response for any individual colony is to segregate its investment in male and female reproductives in time, with males being produced first and queens second. The optimal colony-level solution would not be evolutionary stable at a population level because drones and queen would never co-occur and all colonies would have zero mating success if every colony adopted it. Selection to optimize the timing of reproduction in temperate honey bees must therefore be constrained by the need to coordinate the production of reproductives with other colonies in the population. In empirical observations, honey bee colonies do not segregate investments in drones and queens as the first model scenario suggests; instead drones are maintained throughout late spring and summer before and after swarming occurs^19^.

Under our second model scenario, we asked how the optimal reproductive timing changes when we assume the whole population is adopting the same strategy as the focal colony; under this assumption, the maximum queen availability occurs when the focal colony swarms. When all colonies mirror the focal colony, we find the optimal timing of swarming shifts earlier and drones persist over a longer period. In this scenario, the timing of drone and swarm production is a much closer match to empirically observed data. These results suggest that the selective pressures shaping the timing of reproductive investment may be best understood as a coordination game similar to the Battle of the Sexes^20^. In this game, the action with the highest payoff for males differs from the action with the highest payoff for females. However, both sexes receive the lowest payoff if they fail to coordinate and take different actions. If drones and queens did not need to find each other, the external constraints of seasonal resource availability lead to different optimal timings for their production. However, the additional constraint of coordination leads to a compromise that would be suboptimal based on external resource constraints alone.

Because the present model focuses on optimizing the behavior of an individual colony, it does not capture the evolutionary dynamics that occur if every colony is allowed to adopt any strategy of reproductive timing. In reality, there may be other types of equilibria other than all colonies adopting the same strategy, which would require a game theoretic model to reveal. By assuming all colonies in the population have the same set of constraints, the present model also ignores inter-colony variability, which, in reality, likely impacts reproductive decisions. For instance, larger colonies generally invest more in drones than small colonies because they can better afford to support them^21^. It is possible that colonies adopt condition-dependent strategies of reproductive investment, which would affect the mating success of other colonies^22^. This variability does not alleviate the need for coordination between colonies in the timing of male and female reproductives; rather colonies that overproduce drones relative to the population average will experience selection to coordinate the timing of their drone production to match the timing of queen production in colonies overproducing females. However, ignoring variability may partly explain the slight mismatch between the empirically measured drone timing and that predicted by our model.

The current model allows us to consider ultimate selective pressures governing how a honey bee colony could globally optimize its resource allocation among drones and workers to maximize its annual reproductive success if workers have perfect knowledge of resource conditions and of current and future behavior of all members of the colony. In reality, workers operate on local and likely imperfect information about the colony’s internal state and external conditions. Workers may therefore be under selection to locally maximize current reproductive success without regard to how current behavior affects future allocation decisions. To understand how locally triggered decisions would differ from globally optimal outcomes, however, would require consideration of the quality of potential proximate cues that might allow local information to provide insight into the same externalities that govern the global system. Regardless, there will still be an important coordination constraint acting on non-optimal, locally maximal outcomes.

We have employed a novel computational method for examining optimal reproductive timing in swarm-founding social insects. Our approach makes testable predictions about the optimal timing of resource allocation on colony growth and reproduction, given a limited season for resource gathering. Our results also reveal a fundamental, but previously overlooked, tradeoff between the energetically optimal time to invest in males in terms of maximizing overall colony growth and the need to invest in males in ways that shape colony growth such that that drone and swarm (hence queen) production will co-occur.

Though not the focus of the present work, this method also provides a novel methodology for testing predictions about investment sex ratio in swarm founding social insects. This approach has the advantage of not needing to account for what fraction of workers should be considered indirect investments in males versus investments in the swarm. Instead, worker number is optimized directly, considering its effect on both male and female elements of reproductive success. A similar computational approach could also be applied more broadly to organisms that reproduce both sexually and by fission at the organismal level. Future work will hopefully apply this linear programming approach to understanding the timing of life history events in a variety of taxa, and a diversity of forms of reproductive investment.

## Methods

We develop a linear programming model where the objective function to be maximized is colony fitness. We model a single colony for one season, where the colony is the decision-making agent and the variables to be manipulated include the number of worker eggs and drone eggs to rear each day, and the number of adult drones to eject from the colony each day. All other variables are deterministic, linear functions of those three variables (Table 1). Because colony activity occurs on a daily cycle, we use a series of discrete time difference equations to model colony growth. We assume that:

- The colony’s ability to rear new bees is limited by the amount of food available to feed them and by the queen’s egg laying capacity.
- Brood rearing is not limited by nurse bees because the colony appropriately allocates adult workers between nursing and foraging^23^.
- Adult workers have a net positive energy contribution and adult drones a net negative contribution, since they take energy to feed.
- Any energy not used each day is stored as honey. The colony can use more energy than it produces each day as long as there is enough honey to make up the deficit.
- Colonies produce one prime swarm each year and one afterswarm *g* days later.
- Colonies start with an average spring size *B*_*1*_ and workers live an average number of days *l*_*w*_.
- Drones live until the workers eject them from the colony but experience reproductive senescence with age.
- Drone mating success depends on the number of available queens and all drones have an equal chance of mating.

**Table 1:** Full list of model variables.

Variable name	Variable meaning
**E(t)**	Number of drone eggs laid in colony on day *t*
**D(t,s)**	Number of *s* day old drones in colony on day *t*
**K(t,s)**	Number of *s* day old drones ejected from colony after day *t*
**H(t)**	Number of worker eggs laid in colony on day *t*
**B(t)**	Number of adult workers in colony on day *t*
**S(1,t)**	Number of workers in prime swarm on day *t*
**S(2,t)**	Number of workers in afterswarm on day *t*
**G(d,t)**	Number of worker eggs laid in swarm *d* on day *t*
**F(t)**	Amount of food (in g honey) stored in parent colony on day *t*

Although multiple theories exist about what proximal cues cause colonies to swarm^24, 25^, we assume the ultimate cause of swarming is the colony reaching reproductive stability, i.e. where the amount of food available to rear new workers exceeds the queen’s capacity to lay them^26^. To determine when it is optimal for the colony to swarm, we define the swarm date as t* and solve the model to find the optimal value of the objective function. We then run the model for all possible values of t* within 1..T. We define the optimal swarm date as the value of t* which maximizes the optimal value of the objective function.

### Model constraints

If we define *D*_*t,s*_ as the number of *s* day old drones in the colony on day *t* and *E*_*t*_ as the number of drone eggs laid on day *t*, then ${D}_{t,1}=\,{E}_{t-{e}_{d}}$ where *e*_*d*_ is the number of days for a drone to develop to adulthood. We define ${D}_{t,s}={D}_{t-1,s-1}-{K}_{t-1,s-1}$ where *K*_*t,s*_ is the number of *s* day old drones ejected from the colony after day *t*. For each day *t*, ${K}_{t,s}\le \,{D}_{t,s}$, i.e. the colony cannot kick out more drones than it has. If *B*_*t*_ is the number of adult workers in the colony on day *t*, *H*_*t*_ is the number of worker eggs laid on day *t*, *e*_*w*_ is the number of days for a worker to develop to adulthood, and *l*_*w*_ is the average lifespan of a worker (from egg to death), then ${B}_{t}={B}_{t-1}+{H}_{t-{e}_{w}}-{H}_{t-{l}_{w}}$. If we define t* as the day on which the first swarm issues from the parent colony, $({t}^{\ast }+g)$ as the day the second swarm issues from the colony, and *sf*_*d*_ as the fraction of workers that go with swarm *d*, ${B}_{{t}^{\ast }}=(1-s{f}_{1}){B}_{t-1}+{H}_{t-{e}_{w}}-\,{H}_{t-{l}_{w}}$ and ${B}_{{t}^{\ast }+g}=(1-s{f}_{2}){B}_{t-1}+{H}_{t-{e}_{w}}-\,{H}_{t-{l}_{w}}$.

We assume for all days *t*, ${B}_{t}\ge {B}_{{\rm{\min }}}$ where *B*_*min*_ is the critical size for the colony to stay alive. The colony energy budget is a function of the number of workers in the colony and the amount of energy used to rear workers, rear drones, and feed drones. For each day *t*, ${F}_{t}={F}_{t-1}+{n}_{t}{B}_{t}-m\sum _{{\forall }_{s}}{D}_{t,s}-p{H}_{t}-o{E}_{t}$ where *F*_*t*_ is the amount of honey stored in the colony, *n*_*t*_ is the net daily energy contribution of a worker, *m* is the daily energy consumed by a drone, *p* is the energy needed to rear a worker until eclosion, and *o* is the energy needed to rear a drone to eclosion. The net daily energy contribution per worker is an inverse parabolic function of day *t* such that: ${n}_{t}=\frac{{n}_{int}-\,{n}_{y}}{{n}_{x}^{2}}{(t-{n}_{x})}^{2}+{n}_{y}$ (see Supplementary Information Table S1 and Fig. S9). In addition, the honey stored in the colony cannot exceed a maximum storage capacity *v*, i.e. ${F}_{t}\le v$. The total number of worker and drone eggs laid each day cannot exceed the maximum laying rate of the queen, *r*_*max*_, i.e. ${H}_{t}+{E}_{t}\le {r}_{{\rm{\max }}}$. For *q* days after swarming, *H*_*t*_ and *E*_*t*_ = 0^1^. On the day of swarming, the colony must meet reproductive stability, i.e. $\frac{{n}_{{t}^{\ast }}{B}_{{t}^{\ast }}-\,m{D}_{{t}^{\ast }}-o{E}_{{t}^{\ast }}}{p}\ge {r}_{{\rm{\max }}}$.

At the end of the active season, the parent colony must be of sufficient size, *B*_*minT*_, and have sufficient honey stores, *F*_*min*_, to survive winter, i.e. ${B}_{T}\ge {B}_{{\rm{minT}}}$ and ${F}_{T}\ge {F}_{{\rm{\min }}}$. Swarms grow at a deterministic rate; their final size depends on their initial size and date of issue. If *S*_*d,t*_ is the number of workers in swarm *d* on day *t* and *G*_*d,t*_ is the number of worker eggs in swarm *d* on day *t*, ${S}_{d,t}={S}_{d,t-1}+{G}_{d,t-{e}_{w}}-{G}_{d,t-{l}_{w}}$ and ${G}_{d,t}=\,{\rm{\min }}({r}_{max},\frac{{n}_{t}{S}_{d,t}}{p})$.

We define *O*_*t,s*_, the expected number of offspring produced by an *s* day old drone on day *t*, as the product of *c*_*t,s*_, the probability of an *s* day old drone mating on day *t*, and *w*_*t*_, the final size of an average swarm issued on day *t*, i.e. ${O}_{t,s}=\,{c}_{t,s}{w}_{t}$. The value *w*_*t*_ is calculated deterministically for each day *t* using the same growth parameters as swarm growth and *c*_*t,s*_ is taken from the empirical literature^19^. Drones are assumed to reach maturity at age 12 days and experience reproductive senescence after maturity, where *c*_*t,s*_ is a linearly decreasing function of *s* ^1^.

### Comparison with/without coordination between sexes

We compared two alternative scenarios in the model. In Scenario 1, the expected drone mating probability on day *t*, *c*_*t,s*_, is proportional to the empirically observed number of queens available on day *t*, as reported by Lee & Winston^19^; in this data set, available queens were present from May to late July with the peak occurring in late May (see Fig. S1). In this scenario, *c*_*t,s*_ is independent of the day the focal colony swarms, i.e. other colonies in the population produce swarms independent of the choices of the focal colony (Fig. S1).

Without any other constraints, we would expect all colonies in the population to evolve toward the optimal swarm date. If the focal colony behaves optimally by swarming on a different day than everyone else, there will be no stable evolutionary equilibrium because other colonies could do better by swarming later or earlier. In Scenario 2, we asked how the optimal times for a colony to swarm, and to produce drones, changes if all colonies in the population swarm on the same day as the focal colony, i.e. if colonies coordinate the production of reproductives. To model this scenario, we assumed the peak value of *c*_*t,s*_ always occurs on day *t*_*d*_, the day the focal colony swarms, while keeping the shape of *c*_*t,s*_ the same as in Lee & Winston^19^.

### Model objective function

The objective to be maximized is the sum of all offspring colonies plus the original parent colony times the size of each colony, where size is a proxy for colony survival probability^19^. The value of each offspring colony in the objective function is discounted by its relatedness to the workers of the parent colony. We assume the workers control the level of investment in drone and worker brood since workers can control which larvae are reared through selective feeding and brood cannibalism^27^. Our objective is therefore defined as: maximize: ${k}_{1}(\sum _{{\forall }_{s,t}}{O}_{t,s}{D}_{t,s})+\,{k}_{3}({B}_{T})+\,{k}_{2}({S}_{1,T})+{k}_{3}({S}_{2,T})$, where *k*_*1*_ is relatedness to workers in drone-fathered colonies, *k*_*2*_ is relatedness among workers in the mother queen-led colony, and *k*_*3*_ is the relatedness to workers in daughter queen-led colonies. For each day *t*, we define t* = *t* and find the optimal solution. Then for all possible values of t*, we define the optimal swarm date to be the value t* which produces the maximum value of the objective function.

## Electronic supplementary material

Supplementary Information
