# Supplementary material for: Coordination Between the Sexes Constrains the Optimization of Reproductive Timing in Honey Bee Colonies
Source: Sci Rep. 2017 Jun 1;7:2740. doi: 10.1038/s41598-017-02878-8 (PMC5453950; doi:10.1038/s41598-017-02878-8)
Supplement: Supplementary file 1 — Supplementary Information [file 41598_2017_2878_MOESM1_ESM.pdf]

Title: Coordination Between the Sexes Constrains the Optimization of Reproductive Timing in Honey Bee Colonies

Authors: Natalie J. Lemanski[1]* and Nina H. Fefferman[2]

[1] Department of Ecology, Evolution, and Natural Resources, Rutgers University, New Brunswick, New Jersey, United States of America

[2] Department of Ecology and Evolutionary Biology, University of Tennessee, Knoxville, Tennessee, United States of America

* Corresponding author

E-mail: Natalie.lemanski@rutgers.edu

Supplementary Information

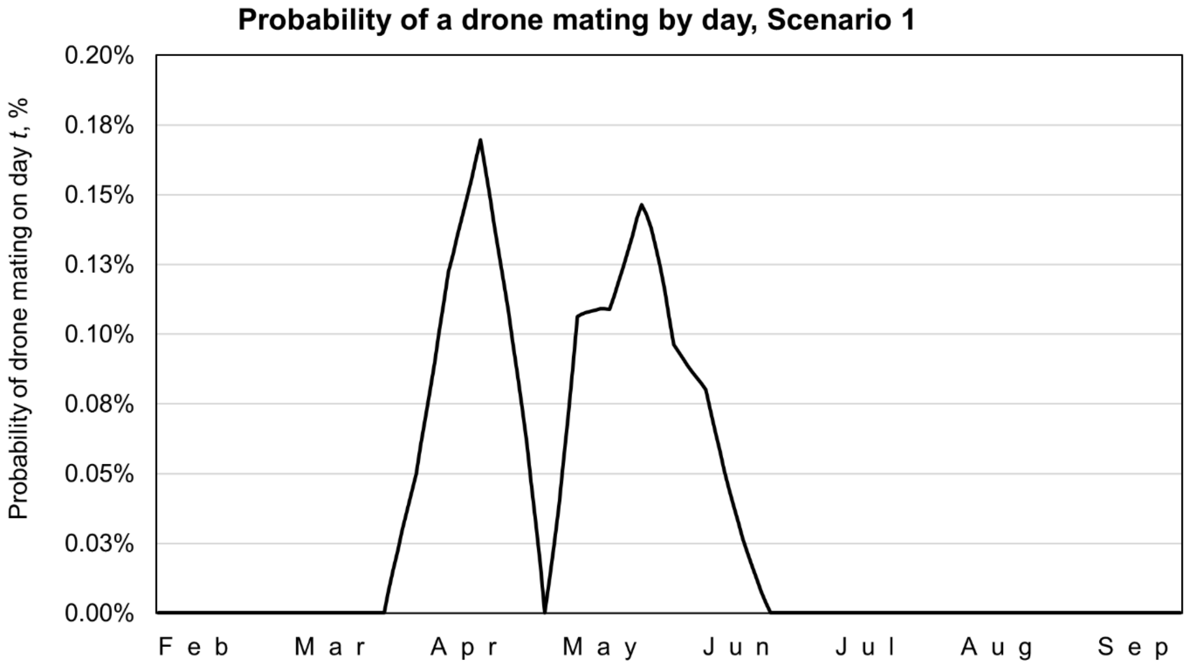

Figure S1. Probability of a drone mating each day based on empirically estimated queen numbers.

Under model scenario 1, we assumed that the probability of a drone mating on each day is a function of the number of queens produced by other colonies in the population on that day, as estimated empirically[1]. The number of available queens has two peaks, one in April and one in late May. We assumed for simplicity that queens are available for mating on the day swarms are produced.

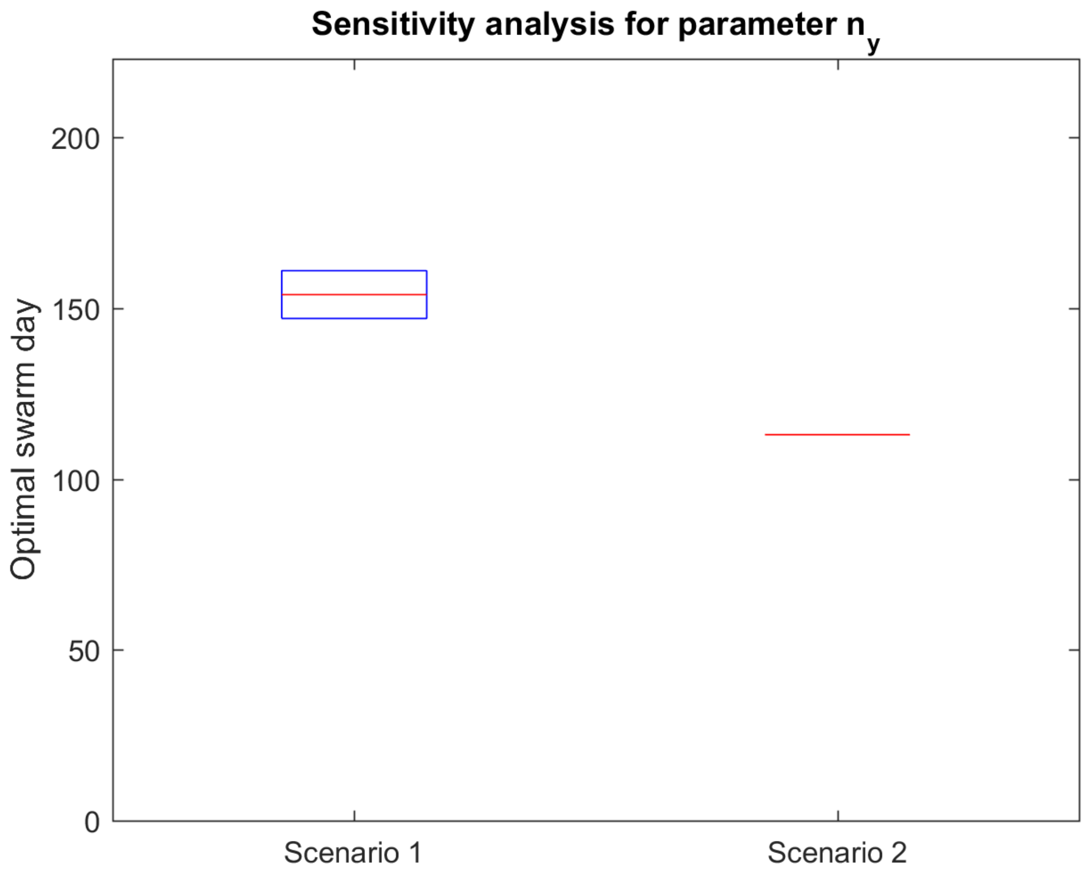

Figure S2. Sensitivity analysis for net worker daily energy contribution.

We performed a sensitivity analysis to examine how our estimate of parameter n_y, the net daily energy contribution of a worker, influences the model outcome. We examined the original value and a range of +/- 10%. The low-end value results in no feasible solution to the optimization problem because worker productivity is too low for the colony to sustain itself.

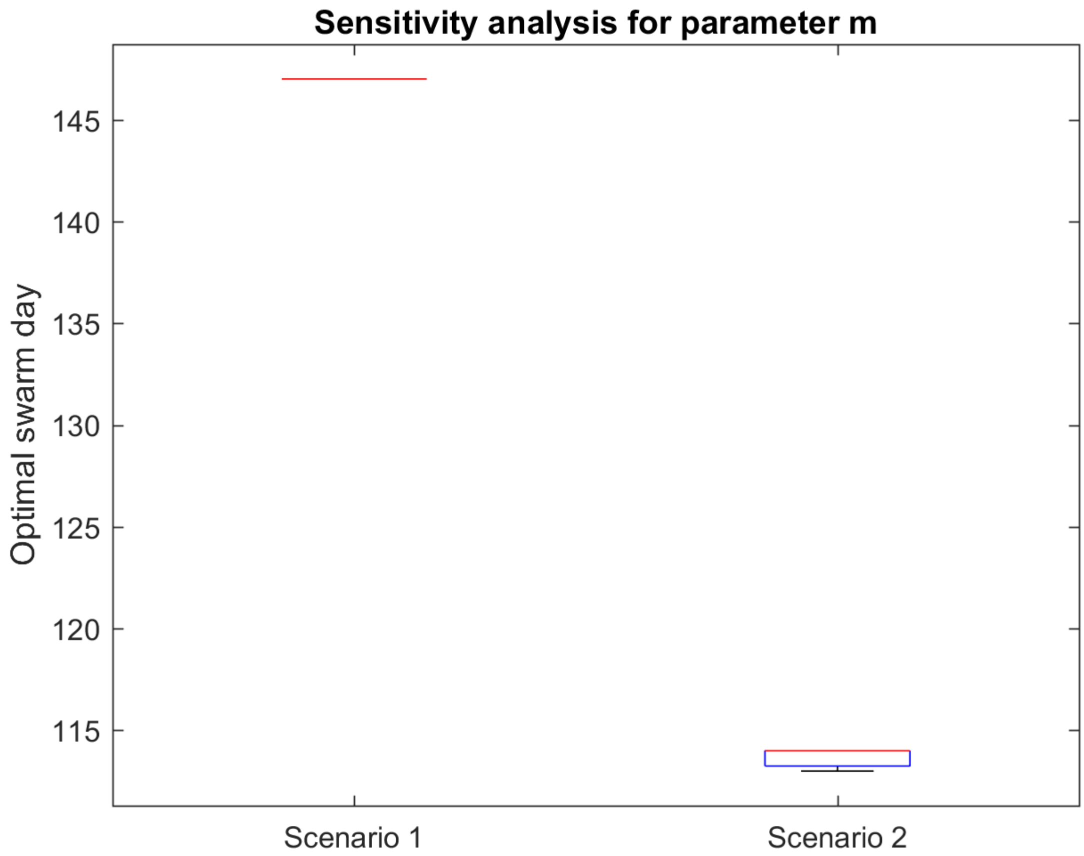

Figure S3. Sensitivity analysis for daily drone energy consumption.

We performed a sensitivity analysis to examine how our estimate of parameter *m*, the daily energy consumption of a drone, influences the optimal timing of swarming under our two model scenarios. We examined the original value and a range of +/- 10%. Within this range, the exact value of *m* has little effect on the optimal swarm time.

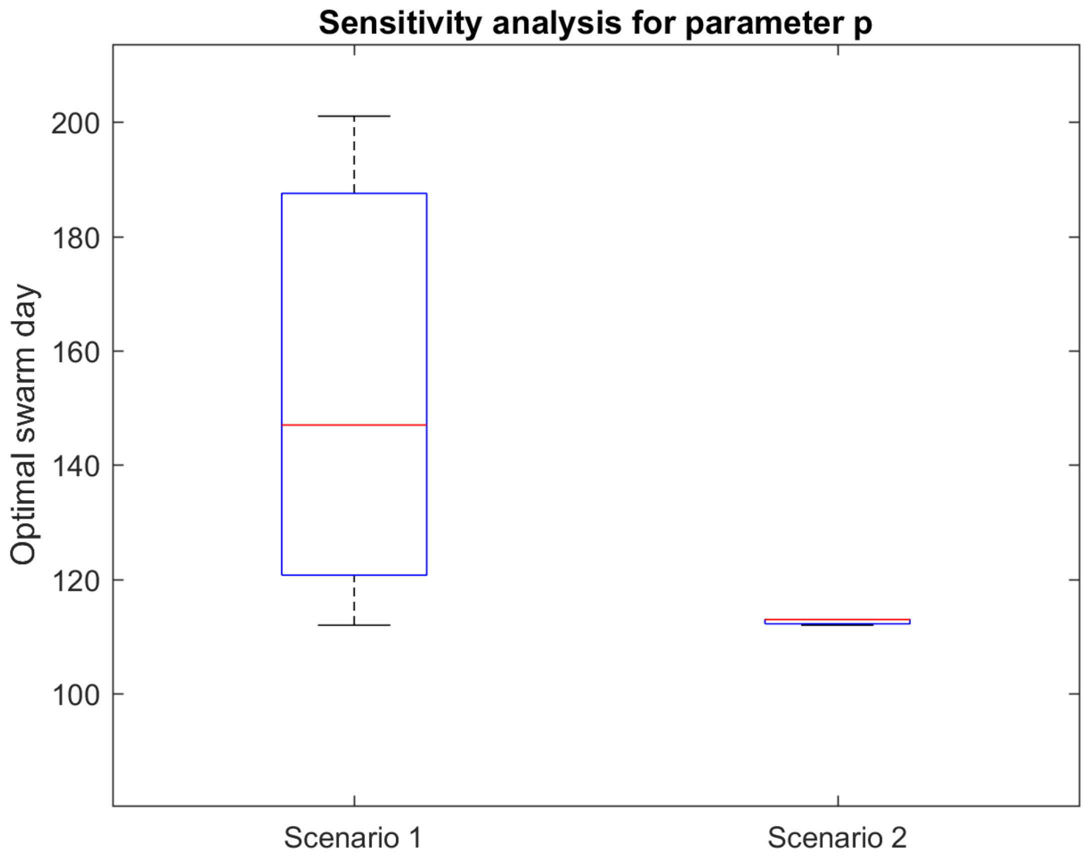

Figure S4. Sensitivity analysis for energy needed to rear a worker.

We performed a sensitivity analysis to examine how our estimate of parameter *p*, the energy needed to rear a worker to adulthood, affects the optimal timing of swarming. We examined the original value and a range of +/- 10%. While, we still find a different optimal behavior between our two model scenarios, the magnitude of the difference does depend on the energetic cost of producing workers.

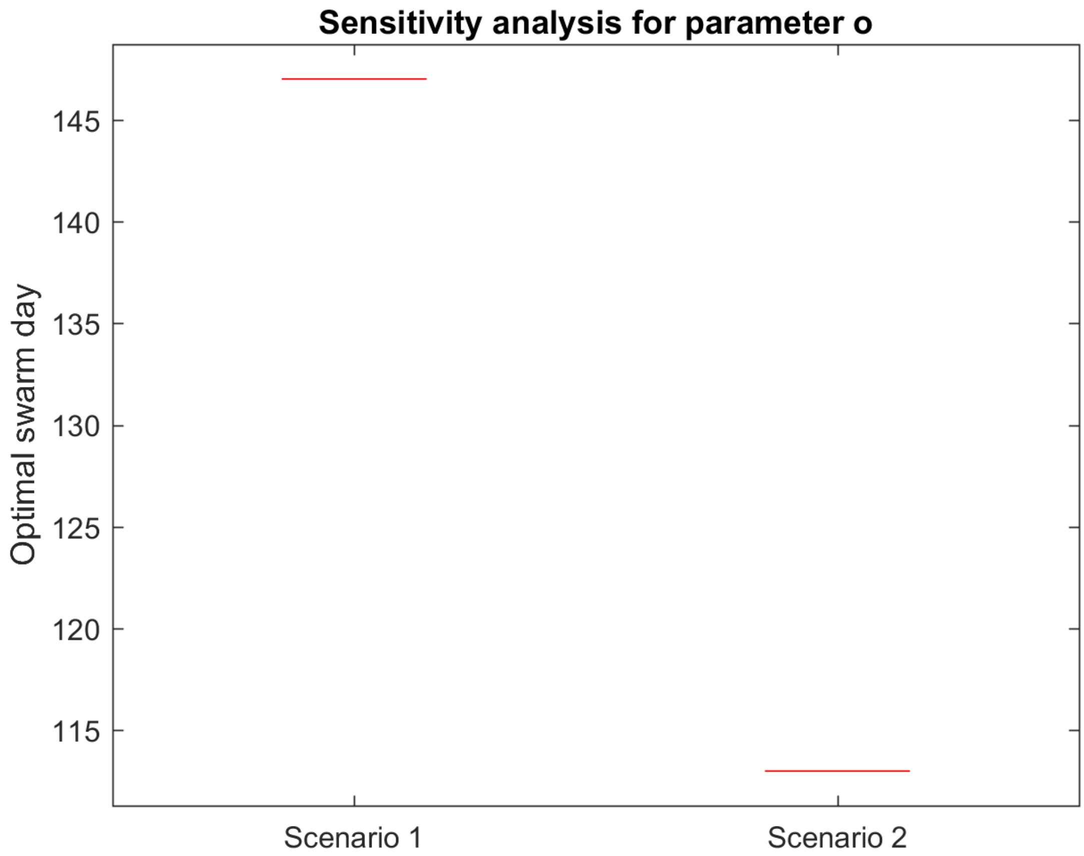

Figure S5. Sensitivity analysis for energy needed to rear a drone.

We performed a sensitivity analysis to examine how our estimate of parameter *o*, the energy needed to rear a drone to adulthood, affects the optimal timing of swarming. We examined the original value and a range of +/- 10%. Within this range, we find little effect of the energetic cost of producing drones on the optimal solution. This makes sense given that the main cost of producing a drone is the need to support it for as long as it remains in the colony; the initial cost to rear a drone to adulthood is relatively small relative to maintenance costs.

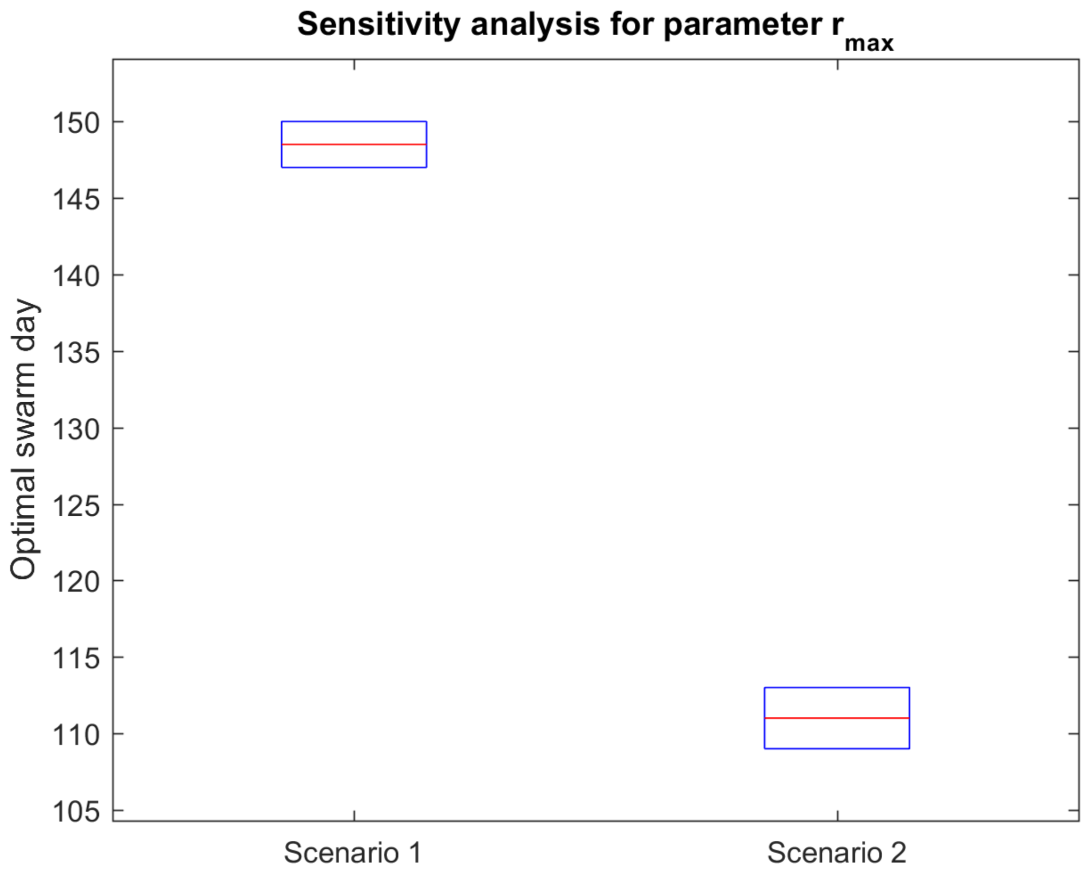

Figure S6. Sensitivity analysis for queen's maximum laying capacity.

We performed a sensitivity analysis to examine how our estimate of parameter r_{max}, the maximum number of eggs the queen can lay per day, affects the optimal swarm date. We examined the original value and a range of +/- 10%. On the low end of this range, the model has no feasible solution. In the parameter range where there is a feasible solution, the exact value of r_{max} has little effect on the optimal swarm date.

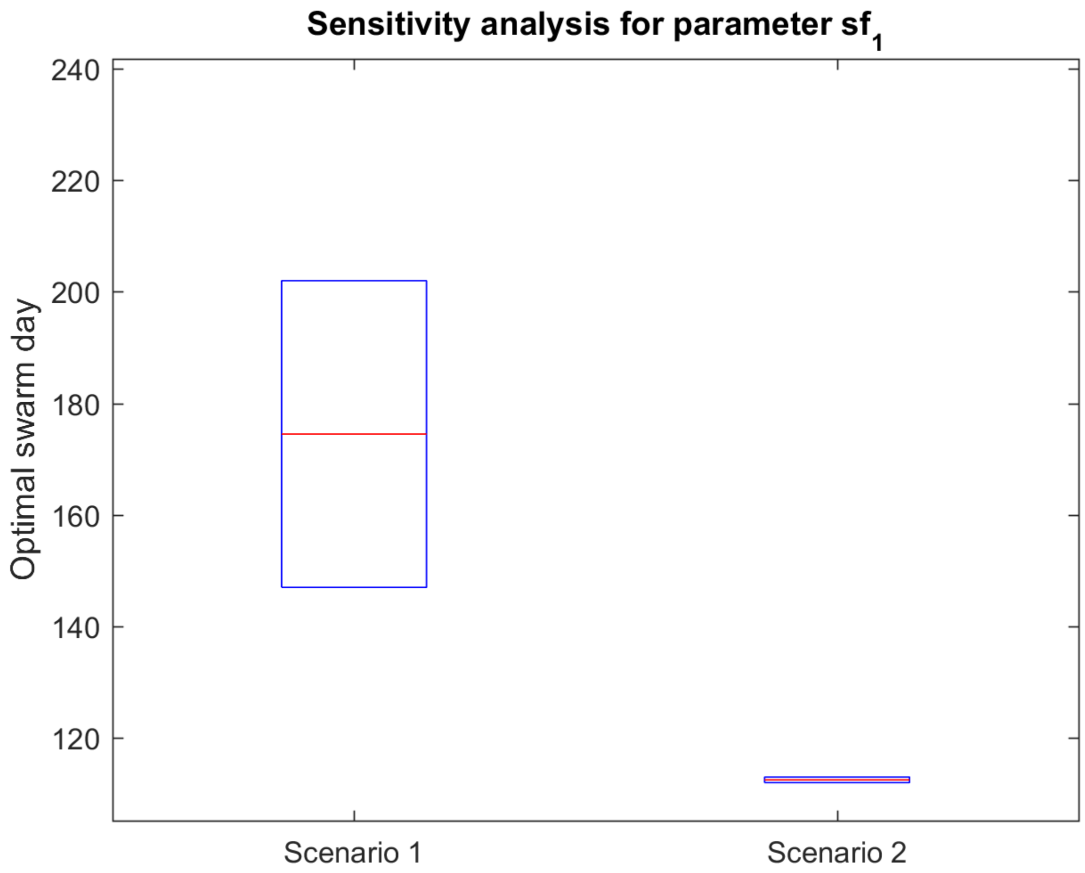

Figure S7. Sensitivity analysis for fraction of colony leaving with swarm.

We performed a sensitivity analysis to examine how our estimate of parameter sf_1, the fraction of the worker population leaving with the prime swarm, affects the model outcome. We examined the original value and a range of +/- 10%. On the high end of this range, the model has no feasible solution because there are not enough workers remaining in the parent colony to sustain it. Within the range where there is a feasible solution, there remains a difference in optimal swarm time between model scenarios 1 and 2, although the magnitude of the difference depends on the size of the swarm fraction.

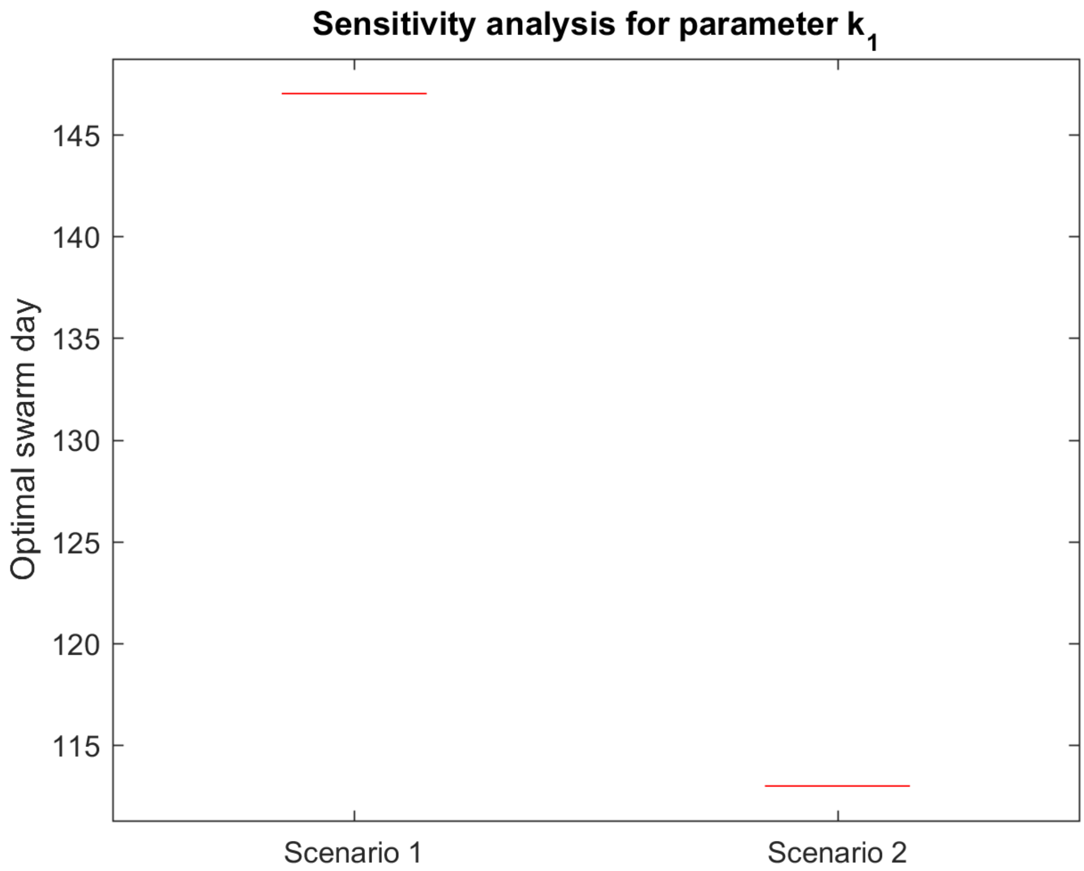

Figure S8. Sensitivity analysis for relatedness of workers to drone-fathered colonies.

We performed a sensitivity analysis to examine how parameter k_1, the relatedness of workers in the original colony to the colonies fathered by drones, affects the model results. We examined a parameter range of +/- 10%; within this range, relatedness had no impact on the optimal time of swarming for either scenario 1 or 2.

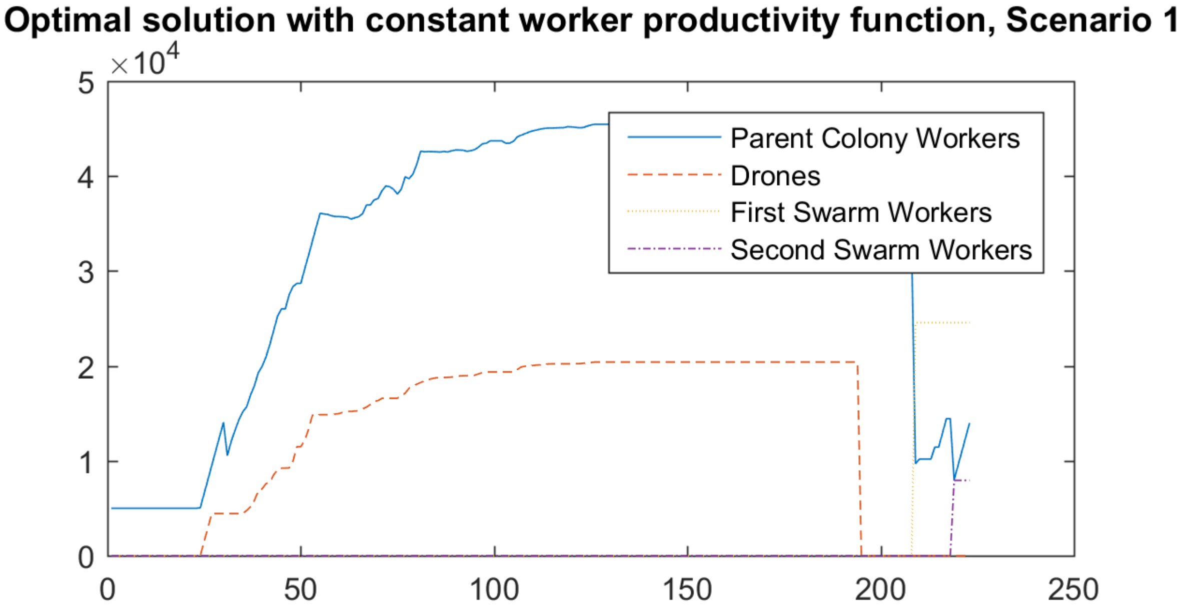

A)

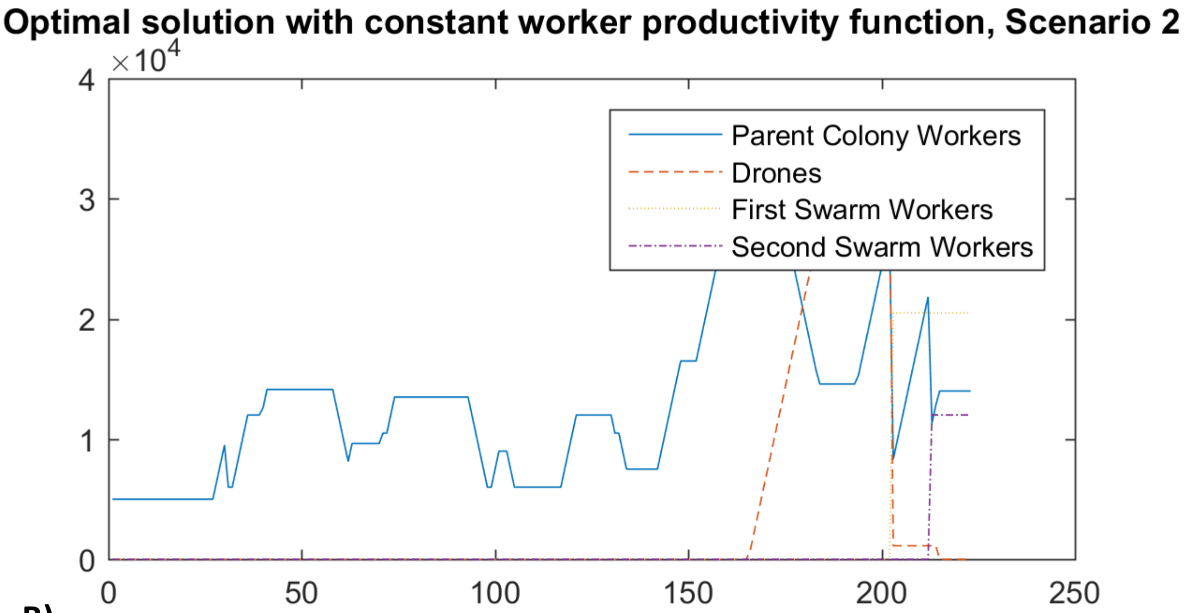

B)

Figure S9. Effect of worker productivity function on optimal swarm time.

We examined the effect of the functional response chosen for n_t, the net daily energy contribution of a worker on day t. In the main paper, we used an inverse quadratic function,

assuming available resources increase throughout the spring and then decrease again in fall. We also examined the effect of constant resource availability (panels A and B). Panel A shows the optimal solution under model Scenario 1, in which we assumed only the focal colony swarms on the optimal day. Panel B shows the optimal solution under model Scenario 2, in which all colonies in the population swarm on the optimal day.

Table S1. Full list of model parameters and their values.

Parameters		Value	Source
**T**	Number of days considered in model	223	Estimated
**n_x**	Worker daily energy contribution x maximum	112	[2,3]
**n_y**	Worker daily energy contribution y maximum	0.0444	[2,3]
**n_{int}**	Worker daily energy contribution y intercept	0.0111	[2,3]
**m**	Daily energy consumption per drone	.0665	[2,3]
**p**	Energy needed to rear a worker to adulthood	.351	[2]
**o**	Energy needed to rear a drone to adulthood	.142	[2]
**e_d**	Days needed for drone to develop from egg to adult	24	[2]
**e_w**	Days needed for worker to develop from egg to adult	21	[2]
**l_w**	Worker life span (from egg to death)	52	[2]
**r_{max}**	Max number of eggs queen can lay per day	1,500	[2]
**v**	Max amount of honey that can be stored in colony at a time	50,000	[4]
**B_{minT}**	Min workers parent colony must have at end of active season	14,000	[2]
**F_{min}**	Min honey parent colony must have at end of active season	20,000	[2]
**B_{min}**	Min workers parent colony must have for all days to remain viable	5,000	[2]
**k_1**	Average relatedness of workers in parent colony to drone-fathered colonies	0.02083	[2]
**k_2**	Average relatedness of workers in parent colony to each other	0.29167	[2]
**k_3**	Average relatedness of workers in parent colony to daughter queen-founded colonies	0.14583	[2]
**w_t**	Final size of an average swarm produced on day t		Calculated

$c_{t,s}$	Probability of an s day old drone mating on day t		[1]
B_1	Initial number of workers in parent colony on day 1	5,000	[2]
F_1	Initial honey stored in parent colony on day 1	5,000	Estimated
sf_1	Fraction of workers leaving with prime swarm	.75	[5]
sf_2	Fraction of workers leaving with afterswarm	.55	[5]
**g**	Number of days between issue of prime swarm and afterswarm	10	[2]
**q**	Days needed for a new queen to start producing eggs	8	[2]

References

1. Lee, P. C. & Winston, M. L. Effects of reproductive timing and colony size on the survival, offspring colony size and drone production in the honey bee (Apis mellifera). *Ecol. Entomol.* **12,** 187–195 (1987).
2. Winston, M. L. *The Biology of the Honey Bee*. (Harvard University Press, 1987).
3. USEPA. White Paper in Support of the Proposed Risk Assessment Process for Bees. 1–275 (2012).
4. Seeley, T. Measurement of nest cavity volume by the honey bee (Apis mellifera). *Behav. Ecol. Sociobiol.* **2,** 201–227 (1977).
5. Rangel, J. & Seeley, T. D. Colony fissioning in honey bees: Size and significance of the swarm fraction. *Insectes Soc.* **59,** 453–462 (2012).
